# Hepatitis B vaccine knowledge and vaccination status among health care workers of Bahir Dar City Administration, Northwest Ethiopia: a cross sectional study

**DOI:** 10.1186/s12879-015-0756-8

**Published:** 2015-01-31

**Authors:** Gedefaw Abeje, Muluken Azage

**Affiliations:** Bahir Dar University, College of Medicine and Health Sciences, School of Public Health, P.O.Box 79, Bahir Dar, Ethiopia

**Keywords:** Hepatitis B vaccine, Bahir Dar City Administration, Health care workers

## Abstract

**Background:**

Hepatitis B infection is a major public health problem in Ethiopia. Health care workers are at increased risk of acquiring hepatitis B infection due to occupational exposure. There is effective and safe vaccine against hepatitis B infection. But many health care workers in developing countries are not vaccinated. There is no study in Ethiopia that describes hepatitis B vaccine knowledge and vaccination status of health care workers. Therefore, this study was done to assess hepatitis B vaccination status and knowledge among health care workers’ of Bahir Dar city administration, Northwest Ethiopia.

**Methods:**

Institution based cross sectional study design was employed from April 1 to 30, 2012. All healthcare workers who were working in Health care facilities of Bahir Dar city administration were the study populations. A total of 374 health care workers were included in the study. Simple random sampling technique was used to select eligible study participants from the list of health care workers. Self administered questionnaire was used to collect data. The completeness of questionnaires was checked every day by facilitators and principal investigators. Data were entered and analyzed with statistical package for social sciences version 16.0 software.

**Result:**

In this study, 64.7% of respondents perceived their risk of acquiring hepatitis B infection very high or high. Only 52% of the respondents were knowledgeable about hepatitis B infection. In this study, only 62% of health care workers were knowledgeable about hepatitis B vaccine. From the total of 370 respondents, only 20(5.4%) reported that they took three or more doses of hepatitis B vaccine.

**Conclusion:**

Hepatitis B vaccination status of health care workers in the study area was low. Health care workers’ knowledge about hepatitis B infection and hepatitis B vaccine was also low as all health care workers should be knowledgeable.

## Background

Hepatitis B is a disease caused by hepatitis B virus (HBV), which is transmitted through percutaneous or per mucosal exposure to infectious blood or body fluids, mainly semen and vaginal fluid [1]. It is one of the most serious of the 20 blood borne pathogens which are the major threat to health care workers (HCWs) [2,3]. It is a major problem because it can cause chronic infection, resulting in cirrhosis of the liver, liver cancer, liver failure and death. Moreover, extra hepatic lesions occur in other organs of the body particularly kidney by deposition of immune complexes as result of this infection [4]. Persons with chronic infection also remain a carrier for HBV transmission [1].

HBV accounts for an estimated 360 million chronic infections with about a million who die each year from chronic liver diseases [5,6]. Most persons who become chronic carriers of the virus live in Asia and Africa [7].

Studies conducted among different segments of the population in Ethiopia showed that hepatitis B virus is a major public health problem in the country. A study conducted in Addis Ababa to investigate sero-epidemiology of hepatitis B virus estimated that the prevalence of hepatitis B virus infection was 7% [8].

The prevalence of hepatitis B virus infection was estimated 2.1% in a study done among voluntary blood donors of Jimma University specialized teaching hospital [9]. Studies among voluntary testing and counselling clients in Shashemene General Hospital and Addis Ababa showed that 5.7% of voluntary testing and counselling clients had hepatitis B virus [10,11].

HCWs are at risk of acquiring blood borne disease including HBV due to occupational exposure to blood and body fluids [12-14]. The World Health Organization (WHO) estimated that, of the 35 million HCWs worldwide, 3 million experience percutaneous exposures to blood pathogens each year, of these 2 million are exposed to hepatitis B virus [13-16].

A study conducted among medical waste handlers in Gondar town governmental health institutions revealed that 6% of medical waste handlers had hepatitis B virus in their blood [17].

A study done among HCWs and non HCWs in Ethiopia showed that of the 110 HCWs and 110 non HCWs, hepatitis B virus was detected in 8 (7.3%) and 1 (0.9%) of health care and non health care workers, respectively [18].

HBV can be prevented by practicing standard precautions such as regular personal hygiene; use of protective barriers; and by proper disposal of sharps, body fluids, and other clinical wastes in health care institutions [6,13,14,19]. Moreover, after exposure to blood or body fluids, post-exposure prophylaxis can be administered as a combination of passive immunization with hepatitis B immunoglobulin and vaccination with hepatitis B vaccine [6]. However, the most cost-effective method to prevent and control hepatitis B is through pre-exposure vaccination and compliance of standard precautions [6]. The vaccine has been found to be safe and effective and can protect one for a lifetime [6].

However evidences show that there is knowledge gap among HCWs about hepatitis B vaccine. A study done to assess knowledge of HB vaccination and route of transmission among Pakistan medical students revealed that 85% of the students were aware of availability of the vaccine. In this study, 76% of participates did not have any knowledge about post exposure prophylaxis for hepatitis B [20].

In a study done in South Africa, only 1.8% of HCWs scored high level of knowledge for hepatitis B vaccine. In this study, only 74.5% showed positive attitude towards HBV vaccination. Of the total respondents, 72% of HCWs had received one or more doses of hepatitis B vaccine but only 61.2% of those vaccinated had received all 3 doses of the vaccine [21]. A study done in Paris on HCWs revealed that 69% of the respondents knew the presence of HB vaccination [22].

Studies in different countries showed different findings on HB vaccination status. Studies in Sweden, Pakistan, Turkey, Paris and South Africa showed that 39.8%, 37.2%, 55.8%, 93% and 19.9% of HCWs respectively received three doses of hepatitis B vaccine [22-26].

A safe and effective vaccine against HBV is available throughout the world, yet many HCWs in resource poor countries remain at risk because they are not vaccinated against HBV [27]. The reason for not being vaccinated may be lack of knowledge about the vaccine. In Ethiopia, there are no studies conducted on knowledge of hepatitis B vaccine among HCWs. This study was therefore done to assess knowledge and vaccination status of hepatitis B among HCWs’ Bahir Dar city administration, Northwest Ethiopia.

## Methods

Institutional based cross sectional study design was employed from April 1 to 30, 2012 to assess knowledge of hepatitis B and vaccination status among HCWs of Bahir Dar city administration. Bahir Dar town is the capital city of Amhara National Regional state which is located 565 kms Northwest of Addis Ababa, Ethiopia (Amhara regional Health Bureau. 2011 annual report. unpublished). According to Central Statistics Authority (CSA) report of the 2007 population census, the total population of the town was estimated to be 180,174 (87,160 males and 93,014 females [28]. There were two hospitals (one governmental referral hospital and one private hospital), 8 health centers (4 in urban, 4 in rural), 4 governmental clinics, 2 nongovernmental clinics, 34 private clinics and 10 health posts in the study area during the study period. According to the Bahir Dar city administration Health office report of 2011, there were a total of 464 HCWs in Bahir Dar city administration at the time of study (Bahir Dar City administration health office. 2011 annual report. unpublished).

All HCWs who were working in health care facilities of Bahir Dar city administration were the study populations. Those HCWs (health assistants, all types of nurses, health officers, medical doctors, dentists, and laboratory technologist) who were involved in direct health care service for clients and patients were included in this study. Students who were on practical attachment at healthcare facilities were excluded from the study.

Sample size of this study was determined using single population proportion formula by considering 95% level of confidence, 5% margin of error and proportion of HCWs who were vaccinated with HBV vaccine 61.2% [21]. Adding 10% none response rate, the final sample size was 403.

Simple random sampling technique was used to select eligible study participants from the list of city administration health offices. Self administered questionnaire was used to collect data from HCWs. The questionnaire had three parts; the first part contains socio-demographic characteristics of HCWs, the second part was knowledge of HCWs on hepatitis B vaccine. Level of knowledge of HCWs was categorized based on the mean value; those who scored below the mean were classified less knowledgeable and those who scored above the mean were classified knowledgeable. The third part was about hepatitis B vaccine status of HCWs. The completeness of questionnaires was checked every day by supervisors and principal investigators on each day of data collection. After checking for consistency and completeness, the supervisors submitted the filled questionnaires to the principal investigator. The collected data were double entered by principal investigator to verify whether the data was properly entered or not by data clerk. Data were entered and analyzed with SPSS version 16.0 software.

Ethical clearance was first obtained from the Ethical Clearance Committee of Bahir Dar University. Permission to conduct the study was taken from Bahir Dar City Administration Health Office and Amhara Regional Health Bureau and written consent was taken from the study participants. Privacy and confidentiality were maintained throughout the study period; each questionnaire was number-coded without any personal identification.

## Results

### Socio demographic characteristics of HCWs

The mean age of the respondents was 29.59 (±6.74) years. The mean number of work experience among study participants in years was 7.7 with a standard deviation of 7.4 years. Majority of the respondents (55%) were nurses (Table 1).

**Table 1:** **Socio demographic characteristics of health care workers at Bahir Dar city administration health facilities, August 2012**

**Variable**	**Frequency**	**Percent**
**Sex** (n = 340)		
• Male	145	42.6
• Female	195	57.4
**Occupation** (n = 373)		
• Physician	13	3.5
• Health office	33	8.8
• Nurse	205	55.0
• Midwife	18	4.8
• Lab technician	77	20.6
• Other	27	7.2
**Marital status** (n = 370)		
• Single	135	35.7
• Married	223	60.3
• Widowed	5	1.4
• divorced	10	2.7
**Department of work** (n = 343)		
• Emergency room	33	9.6
• Outpatient department	125	36.4
• Delivery room	32	9.3
• Operation room	8	2.3
• Ward	32	9.3
• Other	113	32.9

Numbers in () correspond to the number of answers given.

### Perceived risk of acquiring hepatitis B infection among HCWs

Participants were asked to rate their perceived risk of acquiring hepatitis B infection. From the total 354 participants who responded to this question, 166 (46.9%), 63 (17.8%), 72 (20.3%), 25 (7.1%) and 18 (5.1%) respondents rated their risk of acquiring hepatitis B infection very high, high, medium, low and very low respectively. Ten (2.8%) respondents reported that they did not know whether they were at risk or not. From 374 HCWs who answered the question about history of occupational exposure, 335 (89.6%) had positive history (Table 2).

**Table 2:** **History of occupational exposure among health care workers of Bahir Dar city administration, August 2012**

**Question**	**Yes**
	**Number (%)**
History of exposure to blood or body fluids on intact skin (n = 369)	315 (85.4)
History of splash of blood or body fluids to eye or mouth (n = 370)	256 (69.2)
History of splash of blood on cuts or unprotected skin (n = 368)	211(57.3)

Numbers in () correspond to the number of answers given.

### Knowledge of HCWs about hepatitis B infection

Respondents were asked 10 questions about hepatitis B infection. The maximum and minimum scores were 10 and 5 respectively. The mean knowledge score of the respondents about hepatitis B infection was 7.6 with standard deviation of 1.27 and range of 9. About 52% of the respondents scored above the mean knowledge score about hepatitis B infection. About 95% of the respondents correctly responded that hepatitis B is more infectious than HIV. Similarly, about 82% agreed that hepatitis B infection is more common in Sub Saharan Africa (Table 3).

**Table 3:** **Knowledge of hepatitis B infection among health care workers of Bahir Dar City Administration, August 2012**

**Question**	**True**
	**Number (%)**
One can get hepatitis B infection through needle stick injury (n = 375)	347 (92.5)
Hepatitis B infection can be prevented by vaccination (n = 365)	347 (95.1)
Hepatitis B virus can be found in semen or vaginal fluid of infected person (n = 370)	325 (87.8)
Hepatitis B infected person may be asymptomatic for long time (n = 372)	323 (86.8)
Every person exposed to hepatitis B virus will develop acute hepatitis immediately (n = 359)	169 (47.1)
Hepatitis B virus is highly infectious (n = 370)	355 (95.9)
Only small proportion of the world population is infected with hepatitis B virus (n = 356)	97 (27.2)
Hepatitis B virus mainly affects liver (n = 372)	359 (96.5)

Numbers in () correspond to the number of answers given.

### Knowledge of HCWs about hepatitis B transmission and control

Respondents were asked about hepatitis B prevention and control mechanism. Table 4 below shows the responses of HCWs about hepatitis B infection and control mechanisms.

**Table 4:** **Knowledge of hepatitis B infection prevention and control measures among health care workers of Bahir Dar city administration, August 2012**

**Questions**	**Yes**
	**Number (%)**
Hepatitis virus can be transmitted from one person to the other through**	
Sharps injury	340 (91.2)
Blood donation from infected person	342 (91.7)
Sexual intercourse with infected person	288 (77.2)
From mother to child during pregnancy	247 (66.2)
Feaco oral	78 (20.9)
Polluted water	37 (9.9)
Transmission of hepatitis B infection can be prevented by**	
Vaccination	336 (90.3)
Proper disposal of sharps	313 (84.1)
Avoiding multiple sexual partner	212 (57)
Avoiding drinking contaminated water	36 (9.7)
Avoiding uncooked food	27 (7.3)
Using glove	270 (72.6)

Numbers in () correspond to the number of answers given, **multiple responses were there.

### Vaccination status

Of the total respondents, 370 responded to the question whether they were vaccinated or not at the time of interview. Only thirty seven (10%) respondents reported that they received one or more doses of hepatitis B vaccine. From these, only 20 (54%) received three or more doses which was only 5.4% of the total HCWs. Among 333 respondents who were not vaccinated, 201(60.36%) and 133 (39.93%) reported that the vaccine was not available and costly respectively.

### Knowledge about hepatitis B vaccine

Respondents were asked fourteen item questions to assess their knowledge about hepatitis B vaccine. The maximum and minimum score for these knowledge questions were 14 and 5 respectively. The mean knowledge score for hepatitis B vaccine was 8.85 with standard deviation of 2.67. About 62% of HCWs scored above the mean knowledge score. In this study, 93(27.6) of the respondents wrongly responded that one or two does of hepatitis B vaccine are sufficient to be fully immunized for an adult (Table 5).

**Table 5:** **Knowledge of hepatitis B vaccine among health care workers of Bahir Dar city administration, August 2012**

**Statement**	**True**
	**Number (%)**
There is effective vaccine to prevent hepatitis B infection (n = 361)	337 (93.4)
Hepatitis B vaccine can be given as post-exposure prophylaxis (n = 346)	172 (49.7)
Hepatitis B vaccine is contra indicated for immune compromised patients (n = 351)	168 (47.9)
Hepatitis B vaccine is effective to treat patients with acute hepatitis B infection (n = 352)	89 (25.3)
Hepatitis B vaccine is highly effective in preventing hepatitis B infection if given within 48 hours after exposure (n = 336)	181 (53.9)
Hepatitis B vaccine should be given to health care workers as part of work place safety (n = 363).	333 (91.7)
Full course of hepatitis B vaccine may give lifelong immunity but for Health professionals, one further booster after 5 years of the first dose is recommended (n = 345)	205 (59.4)
After taking full dose vaccination of hepatitis B, there is no need for a blood test to confirm immunity against hepatitis B (n = 351)	126 (35.9)
Full dose hepatitis B vaccine provides 100% protection for 90% of adults (n = 345)	288 (83.5)
Full dose hepatitis B vaccine protects against HBV for at least 15 years (n = 345)	217 (67.2)
Hepatitis B vaccine causes problems if given to people who are already immune (n = 354)	108 (30.5)
Hepatitis B vaccine is recommended for all health care workers (n = 343)	241 (70.3)

Numbers in () correspond to the number of answers given.

## Discussion

### Perceived risk of acquiring hepatitis B infection

HCWs are at risk of acquiring blood borne disease including HBV due to occupational exposure to blood and body fluids [12-14]. From 374 HCWs who responded to the question whether they had history of occupational exposure to blood or body fluids or not, 335 (89.6%) reported history of occupational exposure. This indicates high level of occupational exposure to hepatitis B and other blood borne pathogens.

In the presence of this high level of occupational exposure to blood and body fluids, about 12.2% of the respondents reported that they are at low or very low risk for hepatitis B infection. Similarly, 10 (2.8%) respondents reported that they did not know whether they are at risk or not for hepatitis B infection. This proportion is high as all HCWs should know that they are at high risk of acquiring hepatitis B infection. These HCWs are less likely to take hepatitis B infection prevention and control measures as they thought that they are at low or very low risk of hepatitis B infection.

### Knowledge of HCWs about hepatitis B infection

Only about 52% of the respondents scored above the mean knowledge score about hepatitis B infection. This is below the expectation that all HCWs should know about hepatitis B infection. This may lead to the conclusion that HCWs were less knowledgeable about hepatitis B infection. About 95% of the respondents correctly responded that hepatitis B is more infectious than HIV. Similarly, about 82% of the respondents agreed that hepatitis B infection is more common in Sub Saharan Africa. About 47% of the respondents wrongly responded that all persons exposed to hepatitis B virus will develop acute hepatitis immediately. This is a big gap that should be bridged as soon as possible. Although they were few (4.9%), some HCWs also wrongly reported that there was no vaccine against hepatitis B infection.

### Knowledge of HCWs about hepatitis B transmission and prevention

Most respondents reported that hepatitis B virus may be transmitted from one person to the other through sharps injury (91.2%) and blood transfusion (91.7%). But few HCWs wrongly reported that hepatitis B can be transmitted through feaco-oral rout (20.9%) and through polluted water (9.9%). Similarly, 9.7% and 7.3% of the respondents wrongly reported that hepatitis B infection can be prevented by avoiding drinking contaminated water and eating uncooked food respectively. These facts also show presence of knowledge gap among the respondents about hepatitis B infection transmission and prevention mechanisms.

### Vaccination status

From the total respondents included in this study, 370 gave response to the question that asked them whether they were vaccinated or not at the time of interview. Only thirty seven (10%) respondents reported that they received one or more doses of hepatitis B vaccine at the time of interview. From these, only 20 (54%) received three or more times which is only 5.4% of the total health care workers.. This is very low as the level of occupational exposure is high among the study participants. Ethiopia is also one of the countries with high level of hepatitis B. Unavailability of the vaccine was the main reason mentioned for not being vaccinated by respondents although it is available in the private clinics in Bahir Dar town.

### Knowledge about hepatitis B vaccine

In this study, about 62% of HCWs scored above the mean knowledge score about hepatitis B vaccine. About 72% of the respondents correctly responded that an adult should take three or more doses of hepatitis B vaccine to be fully immunized. But only about 50% of the respondents knew that hepatitis B vaccine could be given as post exposure prophylaxis. About 25% of the respondents also wrongly responded that hepatitis B vaccine could effectively treat patients with acute hepatitis B infection. Only 59.4% of HCWs knew the necessity of additional booster dose five years after completing the three doses of vaccines. All these figures show presence of knowledge gap about hepatitis B vaccine.

This study tried to assess one of the key areas of public health importance. But because of the cross sectional nature of the study and the small sample size, we were not able to asses factors associated with low knowledge and coverage of hepatitis B vaccine.

## Conclusion

In this study, there were HCWs who believed that they were at low or very low risk for hepatitis B infection. Only 5.4% of HCWs also reported that they received three times or more for hepatitis B vaccine. This is a serious public health scenario and challenge for a country with high prevalence of hepatitis B infection.

Knowledge of HCWs about hepatitis B infection, mode of transmission and hepatitis B vaccine was low. There were misunderstandings about these issues among HCWs. Therefore, the Regional Health Bureau and other concerned bodies should give training on hepatitis B infection, prevention and control, and hepatitis B vaccine for HCWs in Bahir Dar City administration.

## Abbreviations

CSA: Central statistics authority
HBV: Hepatitis B virus
HCWs: Health care workers
Lab: Laboratory
OPD: Outpatient department
OR: Operation room
SPSS: Statistical package for social sciences
VCT: Voluntary counselling and testing
WHO: World Health Organization
